# A Qualitative Descriptive Study of Rural Primary Healthcare Professionals’ Capacity for Disaster Health Management Before and During the COVID-19 Pandemic

**DOI:** 10.3390/ijerph22010126

**Published:** 2025-01-20

**Authors:** Ehmaidy Al qaf’an, Stewart Alford, Holly A. Mack, Ravneet Sekhon, Samuel Gray, Kiara Song, Katie Willson, Glynn Kelly, David Lim

**Affiliations:** 1 Centre for Improving Palliative, Aged and Chronic Care Through Clinical Research and Translation (IMPACCT), Faculty of Health, University of Technology Sydney, Sydney 2007, Australia; 2 Kaplan Business School, Kaplan Australia, Brisbane 4000, Australia; 3 School of Health and Medical Sciences, University of Southern Queensland, Brisbane 4000, Australia; 4 Central and Eastern Sydney PHN, Sydney 1460, Australia; 5 College of Medicine and Public Health, Flinders University, Adelaide 5042, Australia; 6 Medical School, The University of Queensland, Brisbane 4072, Australia

**Keywords:** disaster health management, rural health, general practitioners, COVID-19, primary healthcare

## Abstract

Introduction: Rural areas face additional challenges in preventing, preparing for, responding to, and recovering from disasters. This study aimed to understand how rural primary healthcare professionals (PHCPs) perceive their roles, involvement, and capacity in disaster health management. Methods: For this qualitative descriptive research, semi-structured interviews were carried out with convenience and purposive samples of rural PHCPs before and during the COVID-19 pandemic. Open, axial, and selective coding were employed to analyse the data inductively. Results: Five interviews were conducted before the pandemic, and ten interviews were conducted during the second and third waves of the COVID-19 pandemic in Australia. The themes identified were similar between the two periods. Rural PHCPs were underutilised due to a lack of awareness of their capacity and a lack of infrastructure and support for greater involvement. Conclusions: Rural PHCPs can be better integrated and supported in readiness for a whole-system response to future disasters. This study recommends empowering PHCPs in disaster management to promote the health and continuity of care in rural communities.

## 1. Introduction

Disaster management in rural and remote primary healthcare settings is a crucial component of public health that tackles the particular challenges communities face in geographically remote areas [1,2]. Rural and remote regions usually face additional challenges in responding to and recovering from disasters, unlike larger centres, where resources and infrastructure are often more readily available [3,4]. Among these difficulties may be the scant population, limited transportation and communication infrastructure, and restricted access to healthcare facilities [5,6,7,8,9]. The term “disaster” lacks a commonly agreed-upon formal definition, but recurring themes highlight the extent to which severe events affect humanity and their capacity to deplete already limited resources [10,11]. Landesman (2005) defines a disaster as a situation of particular scale and breadth that causes harm to people or assets, injury, illness, or death, and that cannot be sufficiently controlled with the help of resources or established processes. Natural, man-made, and hybrid disasters are the three primary classifications into which types of disasters are frequently divided [12]. Earthquakes, floods, bushfires, landslides, tsunamis, storms, and other extreme weather phenomena are examples of natural disasters. Man-made disasters involve transportation or industrial incidents as well as terrorist attacks, explosives, chemicals, toxins, or nuclear substances [13]. Pandemic-related events, such as viruses, pose a threat to public health and fall under the last category of disasters [14]. The Prevention, Preparedness, Response, and Recovery (PPRR) model is a comprehensive framework for managing disasters [15]. Every disaster goes through three cycle phases: the pre-, during-, and post-disaster phases. Pre-disaster phases comprise preparedness and prevention; during-disaster phases are referred to as reaction phases; and post-disaster phases are recognised as recovery [15].

Disasters can inflict severe harm on communities, leaving a trail of destruction that impacts lives, livelihoods, and infrastructure. For instance, the 2022 report lists 387 natural hazards and disasters that were reported by the Emergency Events Database. These disasters claimed 30,704 lives, impacted 185 million people, and caused USD 223.8 billion in economic damages [16]. The harm caused by disasters extends beyond immediate physical damage [17]. Communities may experience disruptions to essential services such as healthcare, education, and transportation, exacerbating existing vulnerabilities. The loss of homes, livelihoods, and critical community assets can have long-lasting social and economic repercussions [18]. Additionally, the psychological toll on individuals, families, and communities can be profound, leading to increased stress, trauma, and mental health challenges [19].

Maintaining and delivering healthcare services during and after disasters is a complex undertaking that requires a multifaceted and adaptive approach [20]. Disasters, whether natural or human-made, can disrupt healthcare infrastructure, strain resources, and increase the demand for medical services [21]. In disaster management, healthcare services during and after disasters are crucial for mitigating the impact on communities [22]. This requires a comprehensive and integrated approach that considers rural and remote areas’ specific needs and constraints [1,23]. Primary healthcare professionals (PHCPs) are the front-line providers of health services in many countries, addressing a wide range of health issues, offering essential services such as triage, treatment and referrals, and are the initial point of contact in the healthcare system. PHCPs include general practitioners (GPs; also known as family physicians), nurses, midwives, community health workers, pharmacists, paramedics, psychologists and other allied health professionals. PHCPs are especially important in rural areas to ensure fair access to healthcare and are often looked upon to provide leadership and guidance during a public health disaster because of their local knowledge and community trust, which enable them to deliver tailored interventions and support, fostering resilience and recovery [1]. By integrating rural PHCPs with public health efforts during a crisis, these providers can enhance the overall effectiveness and efficiency of disaster response and the capacity for disaster recovery, and contribute significantly to the health system’s resilience. Specifically, during the recent COVID-19 pandemic, PHCPs in England, France, Germany, Israel, Spain, the USA, Singapore, Hong Kong and Australia were engaged in distributing COVID-19 vaccinations to increase vaccine adoption and public confidence [24]. However, the role of PHCPs in the disaster preparedness phase can be improved, as evidenced by a few previous studies [25]. PHCPs can play a more active part in disaster management, especially in rural areas that are faced with additional vulnerabilities and challenges that need specialized strategies [26,27]. This study seeks to fill a knowledge gap about the role of PHCPs in rural disaster management through an understanding of their experiences with disaster management.

## 2. Methods

### 2.1. Theoretical Framework

This study utilised the capacity-building theory, which provides a framework that emphasises the importance of building and sustaining the skills, systems, resources, and adherence needed to enhance health in the health sector along interconnected sectors [28,29]. In other words, capacity-building refers to the behind-the-scenes efforts of healthcare professionals to promote and sustain good healthcare in a changing world [28,29]. Three aspects of health capacity-building are health infrastructure or service development; program maintenance and sustainability; and the problem-solving capability of organizations and communities [28]. Health infrastructure or service development is the process of establishing minimum standards in the health sector’s structures, organizations, skills, and resources. Program maintenance and sustainability emphasizes the ability to carry out a specific program over time through an organization’s network. The problem-solving capability of organisations and communities refers to their ability to recognize health problems and formulate suitable decisions by drawing on past experiences or actions [28]. Applying a capacity-building framework in healthcare, especially during disasters, improves the ability of healthcare systems, professionals, and communities to attend adequately to health crises. This study employed the capacity-building framework to understand the importance of ongoing professional training and education; resource allocation and distribution; community engagement; strengthening healthcare infrastructure; data collection and analysis to assist decision-makers; and developing disaster management policies and guidelines. Moreover, capacity-building works to build responsive systems that involve a focus on the processes that support change within and between organizations, leading to systems that value critical problem-solving and leadership across organisations and address health challenges during disasters [28].

### 2.2. Study Design and Setting

A qualitative descriptive study was undertaken to understand the different experiences of rural PHCPs with disaster health management. This methodology is well established and was specifically selected to recognise the subjective nature of the different experiences and applications of health services [8,9]. Semi-structured interviews informed by key themes from the preceding scoping review [1,8] were conducted in two trenches: 2015 (pre-COVID-19; P1–P5) and 2020 (second and third waves of COVID-19; P6–P15).

Participants completed an informed consent form, stating that they voluntarily decided to participate in the study, procedure, or data collection after fully understanding the study goals, risks, benefits, and handling of their information. Interviews were conducted by three investigators in person and via videoconference. Interviews were recorded with permission and transcribed verbatim, with identifiable and re-identifiable data removed before being sent back to participants for member checking to ensure the credibility of this qualitative research. Interviews lasted, on average, 30–45 min and were conducted at the participant’s convenience.

### 2.3. Study Participants and Sample

This study utilised a convenience and purposive sample of rural vocationally registered Australian PHCPs in the Modified Monash Model 2 (regional centres) to 5 (small rural towns) geographical areas, whom had experience with disaster health management to ensure that the study utlised high practical experience and actionable insights critical for improving disaster management practices. Snowball recruiting over a six-month period was performed through university websites, Rural Doctors Workforce Association and general practice networks. Data sufficiency was reached with the final sample size [30]. Prior to performing the study, formal ethics approval was obtained.

### 2.4. Data Analysis

The authors utilised inductive data analysis first, comparing across groups pre- and during COVID-19, before moving to deductive analysis using the capacity-building framework. Using NVivo software (NVivo 14, Lumivero, Denver, CO, USA), four investigators independently read and coded the interviews inductively using open coding, axial coding, and selective coding. This iterative process allowed for the examination of emergent themes and patterns within the data. This methodological approach is flexible yet able to harness the strength of “multiple realities [and] … diverse perspectives” [31] while fostering the understanding and construction of theory; multiple coding, establishing audit trails, and validation by independent researchers were utilised to improve the dependability of the qualitative research rigour. Reflective journals were kept by the researchers, and fortnightly investigators/supervisory meetings were held to extend the confirmability of the research [32].

## 3. Results

Fifteen PHCPs (coded P01–15) were interviewed across rural and regional Queensland and South Australia. As outlined in Table 1, this study deliberately included participants from various professions and geographical locations.

All the participants had experienced disasters other than COVID-19. The diverse disasters encountered by participants were natural (thunderstorms, flash flooding, bushfires), mass-casualty catastrophes, and infectious (measles, swine flu).

### 3.1. Themes

Five overarching themes emerged from the deductive data analysis: the role of PHCPs in rural disaster health management; the participation of PHCPs in decision-making during rural health disasters; the internal and external enablers of PHCP involvement in disaster management; internal and external barriers to PHCP involvement in disaster management; and the impact of COVID-19 on PHCPs’ experience.

#### 3.1.1. Role of PHCPs in Rural Disaster Health Management

Monitoring role: The monitoring approach emphasises the need for flexibility and agility in disaster management while acknowledging the complexity and uncertainty of disaster scenarios. Monitoring the disaster approach involves a systematic procedure used to pinpoint and weigh potential dangers or risks related to accidents, infectious diseases, and natural disasters. In the case of infectious diseases, for instance, PHCPs need to decide the likelihood that frontline employees may contract the flu.

*“… anybody with swine flu is seen completely away from the risk of patients and staff.”* (P2).

*“if the patient is aware that they or we are aware that they are infectious they can wear the mask on, they can be asked to sit in a separate room”* (P4).

Adopting role: The adopting approach refers to the application of the existing disaster management plans. Based on the data gathered, PHCPs adopt the current disaster management plans and strategies to address the shifting conditions of new disasters.

*“We just follow the protocols for patient management”* (P4).

*“You’re a frontline worker in that role so you take command from the command centre”* (P9).

*“We’ve got our own disaster plan and we’ve picked key areas on how it may affect the business and how it runs.”* (P6).

Disaster coordination role: Disaster coordination involves the collaborative efforts of various organizations, agencies, and stakeholders to effectively respond to and manage all aspects of a disaster or emergency. Coordinating disaster includes command and control; communication management; resource management; and logistics and supply chain management. Building the command structures to oversee and guide response actions, such as identifying incident commanders, emergency operations centres, and ground staff, it is attainable to command and control the disaster. For instance, in a natural disaster, PHCPs guided the nearest rural help (paramedics, firefighters, rescue) to the most required location.

*“that helps them triage and integrate with emergency services: police, fire brigade, emergency services.”* (P5).

*“I would speak to my colleagues at work or my colleagues in other practices”* (P10).

*“when it comes to how other agencies and services run their mass casualties and disaster scenarios, it’s helpful to practice with them”* (P11).

In communication management, PHCPs coordinated communication systems to make sure that all stakeholders were informed. For instance, in the event of mass casualty occurrences, PHCPs in remote areas must make sure they notify the closest hospitals of the severity and quantity of casualties.

*“The aim might be transfer to a local hospital or transfer into tertiary hospitals”* (P7).

*“everyone needs to be informed in the similar fashion”* (P13).

*“and then having open communication with people that provide essential services in the whole state.”* (P13).

Resource management involves coordinating the allocation and distribution of resources such as personnel, equipment, medical supplies, food, water, and shelter to meet the immediate needs of affected populations.

*“make you think about how to best allocate staff and resources”* (P6).

*“… thinking about process, thinking about resources”* (P7).

#### 3.1.2. The Participation of PHCPs in Decision-Making During Rural Health Disaster

Specifically, during COVID-19, participants identified two layers of decision-making during rural disaster health management: local or regional decisions made by the rural primary healthcare providers (for example, logistics for setting up vaccination) and decisions made centrally by the health authorities (for example, vaccination schedule). Without being involved in the centralised decision-making process, the rural PHCPs’ contribution to centralised decision-making was restricted to interpreting the edict to staff, patients, and their local communities. However, rural PHCPs were involved in local workplace safety and human resource management decisions.

*“So, this politician is making decision on health and what he thinks about is how he has his general practitioner interaction. That is unfortunate because they think they know but they don’t”* (P7).

*“I can see why it takes time to make a decision, then time for the government to allow the decision to happen”* (P15).

*“You’re a frontline worker in that role so you take command from the command centre, from the captain or supervisor or whatever it is, you don’t get to decide”* (P9).

#### 3.1.3. Internal and External Enablers to PCHP Involvement in Disaster Management

As shown in Table 2, the data indicate that both before and during COVID-19, similar enablers allowed PHCPs to get more involved in disaster management. The enablers that PHCPs mention include higher-level guidance; established communication channels; resources for acute disaster response; moral obligation; digital technology facilitating business continuity; and continuity of care. Higher-level guidance emerged as an enabler for PHCP involvement in disaster management before and during COVID-19. External guidance is useful in facilitating disaster phases. An essential component of higher-level advice is the dissemination of disaster readiness and management policies by authorities and agencies throughout disaster response and disaster-prone seasons. External communication with PHCPs and in-service communication served as enablers in establishing a support network and updating PHCPs with policies and guidelines. The availability of material and human resources was identified as a key enabler for disaster response. This included basic in-service emergency resources, such as Personal Protective Equipment, and additional supplies provided by authorities during disaster response stages. The flexible surge capacity of PHCPs was an enabler that accommodated staff sickness or absence and shared the patient load with nearby PHCPs to meet the increasing demand for primary healthcare services. The moral obligation to uphold ethical norms, fulfil a duty of care, and protect public health served as enablers that allowed PHCPs to be involved in disaster management. Continuity of care was an enabler for PHCPs to deliver patient and community education on disaster management.

#### 3.1.4. Internal and External Barriers to PCHPs Involvement in Disaster Management

Table 3 shows the external and internal barriers to PHCPs’ involvement in disaster management. These include under-utilisation, insufficient resources, a lack of renumeration, and a lack of interest in managing disasters. The under-utilisation of PHCPs’ potential and resources arises from a lack of understanding and recognition of PHCPs’ role during disaster phases. PHCPs experience a sense of disconnection from the planning process as a result of this lack of involvement, which deters PHCPs from participating actively in disaster management. One of the barriers to PHCPs participating in disaster management is insufficient resources. During a crisis, PHCPs encounter difficulties such as a lack of material, human, and financial resources, thereby making it difficult for PHCPs to participate effectively in disaster management. A key barrier to PHCPs’ interest in working during disasters is a lack of remuneration. Like all other professions, PHCPs have financial commitments and duties. Working during disasters often requires more time and effort, which causes PHCPs to lose focus on personal or regular patient care obligations. PHCPs experience financial hardship or uncertainty when they receive inadequate reimbursement for the additional work during disasters, which deters PHCPs from taking part in disaster response operations. The lack of interest acts as a barrier to PHCPs’ involvement in managing disasters. Some PHCPs believe that disaster management is outside the scope of practice or professional interests.

#### 3.1.5. The Additional Impact of COVID-19 on PHCPs Experience

The experience of healthcare professionals in disaster management underwent profound and unprecedented changes before and during the COVID-19 pandemic. Before the pandemic, healthcare professionals operated in a more routine disaster management, with established protocols for patient care and infectious disease management. The focus was primarily on providing routine medical services, preventive care, and addressing common health concerns. However, with the onset of the COVID-19 pandemic, healthcare professionals found themselves at the forefront of an extraordinary public health crisis.

*“around Feb-March when we first started to realise that they were in a very difficult situation”* (P13).

*“since Covid came, there’s a lot of confusion and a lot of times you’ll see that they say one thing in the morning, and in the afternoon another thing”* (P14).

The demands on their expertise, resilience, and adaptability skyrocketed as they faced a surge in critically ill patients, shortages of medical supplies, and the constant risk of exposure to the virus.

*“if you mean a pandemic like COVID, then you would have every clinic and everybody involved because you’re interested in making the clinic run with all the limitations you have”* (P9).

*“We had to totally adjust our practice”* (P10).

*“because of Covid, because waitlists have been so long, by the time that people get to us, people are a lot more unwell”* (P12).

The pandemic introduced new challenges, including the need for the rapid adoption of telemedicine, increased stress and burnout, and the continuous adaptation to evolving scientific knowledge about the novel coronavirus.

*“it was really, really difficult for anybody to get any information from anywhere”* (P9).

*“rather than it being one shock, isolated incident, it’s been kind of an underlying level of anxiety”* (P12).

The experience of primary healthcare professionals during COVID-19 underscored the importance of flexibility, innovation, and collective resilience in navigating unforeseen and complex healthcare challenges.

*“Yes, COVID management, or develop a proper guideline—not only medicine, but non-pharmacological guidelines as well”* (P14).

*“took longer to develop the immunisations and we used novel things for the development”* (P15).

## 4. Discussion

Rural and remote healthcare settings face unique challenges that can significantly impact the accessibility, delivery and continuity of healthcare services [10]. These challenges stem from a combination of geographic, economic, social, and infrastructural vulnerabilities [33]. The rural primary healthcare sector experiences particular difficulties that influence its capacity to effectively plan, prepare for, react, and respond to a disaster [34]. Rural PHCPs must participate in disaster health management due to being close to vulnerable populations and due to their capacity to act quickly in areas with limited resources and the absence of a supportive network. In contrast, non-rural PHCPs are better resourced and well-informed, relying more on systems and networks, which may affect the speed of responses. Rural PHCPS require specific support to improve efficiency in disaster situations.

While primary healthcare has the potential to offer immediate frontline support and attenuate surges in the whole healthcare system during a disaster [1], in our study, rural PHCPs reported having reactive roles in the overall system response, potentially due to a limited understanding of rural primary healthcare capabilities within the healthcare hierarchy and government. There appears to be a sense of disconnect from the disaster planning and preparation processes, and in addition, planning for long-term recovery and restorative actions can be overshadowed by the emphasis on the immediate response during the COVID-19 pandemic.

During disasters, rural PHCPs play active roles in determining the risk to the public, instigating precautionary measures to mitigate risks, providing direct health and medical care, including reducing the demands on acute hospital care during the disaster response phase while offering regional leadership to effect policies and support a whole-system response, and offering continuity of care during recovery from the physical and psychosocial effects of the disaster.

The rural primary healthcare sector has duality in a disaster: whilst it has the potential to reduce disaster damage and the disruption and development of community and system resilience, as a critical healthcare infrastructure, rural primary health must also be protected from poor system integration to promote communities’ health. Promoting rural community health during disasters can be achieved through strategies such as establishing reliable channels to share critical health information during disasters; using digital health options such as telehealth and mobile health clinics to give medical assistance when access to healthcare facilities is impeded [35,36]; and providing mental health support by setting up crisis counselling hotlines. Moreover, PHCPs can strengthen rural community health during disasters by raising awareness of the risks posed by disasters to the local area; promoting preparedness by informing residents of strategies for preparing for the impacts of disasters and taking action; assisting in developing or providing relevant, tailored health resources that are tailored for to community; advocating for the health needs unique to the region; and promoting activities across different settings, not just at the medical practice, but in schools, workplaces, communities centre and clubs [37,38,39,40]. Aside from the perception of underutilisation among rural PHCPs, several changes or recommendations could be made to enhance healthcare provision in rural regions. Expanding access to possibilities for professional growth and training may assist rural health workers in staying updated on medical developments and feeling more confident. Increasing funding for rural healthcare facilities can help improve access to care while reducing the strain on PHCPs. Incentive programs, such as financial bonuses, could attract more qualified staff to underserved areas, addressing shortages and improving service quality. Encouraging collaboration between PHCPs in rural and urban areas may help improve the sharing of resources and information. A more equal healthcare experience, better health outcomes, and easier access to prompt, high-quality care are just a few of the major effects these improvements would have on PHCPs in rural areas.

Globally, PHCPs can help minimize the negative health impacts of disasters by participating at all stages of the disaster management process, raising awareness among international policymakers, designing initiatives that incorporate primary healthcare into broader disaster management plans, and evaluating the disaster readiness in various political, developmental, and cultural settings [41,42,43,44].

We commenced this research prior to the COVID-19 pandemic to understand the capability and capacity of rural primary healthcare in disaster management to inform resource development to support rural PHCPs through the Royal Australian College of General Practitioners and World Organization of Family Doctors; we were able to continue this work during the initial phases of the pandemic, thus enabling us to compare and contrast the findings before and during the pandemic. Whilst our findings are contextualized to Australia, there is nonetheless potential for the results to be transferable to other developed countries with comparable challenges in resourcing rural and regional health services, such as Canada, New Zealand, England and others [4,8,9,35,45]. We believe findings from this study can promote greater discourse on how rural PHCPs can be deployed in disaster management and foster international collaboration in research and training for rural PHCPs, inspiring collaborative regional and international endeavours that foster disaster-resilient and community-centred care. However, for successful implementation, local variables such as infrastructure availability, population density, cultural variety, and healthcare funding models would need to be considered. Thus, while the Australian experience provides valuable insights, extending these results requires careful contextualisation to ensure relevance and effectiveness in other settings.

## 5. Conclusions

Rural PHCPs reported that they were underutilised in formal disaster policymaking during COVID-19. The barriers identified include a lack of understanding by the healthcare hierarchy and government, and the limited infrastructure necessary to support greater involvement. This study recommends that PHCPs be empowered more in disaster management to improve the health of rural communities, as they are knowledgeable about the local residents’ needs. Rural PHCPs can be efficiently used as essential resources in disaster management by defining roles and duties clearly and creating localised health disaster response units; hence, rural residents will gain many benefits from faster disaster response, better readiness, and more resilience. The findings of this study can be employed to address strategic adjustments in training, policy, infrastructure, and resource allocation to enhance the engagement of rural PHCPs in disaster management. The adjustments present opportunities for skill development, recognition, and community trust for PHCPs who reside in remote areas.

## Figures and Tables

**Table 1:** The characteristics of the study participants (*n* = 15).

Location	*n* (%)
Queensland	5 (33.3)
South Australia	10 (66.6)
Primary Healthcare Role Category	
GPs	9 (60.0)
Nurses	2 (13.3)
Paramedics	2 (13.3)
Pharmacists	1 (6.6)
Psychologist	1 (6.6)

**Table 2:** Internal and external enablers for PCHP involvement in disaster management.

Category	Subcategory	Theme
1. Higher-Level Guidance	(1a) External guidance from primary healthcare networks and agencies	Provision of disaster management education and training by primary healthcare networks and agencies. Dissemination of disaster management policies by primary healthcare networks and agencies during disaster response. Dissemination of disaster readiness guidelines by primary healthcare networks and agencies during disaster-prone seasons. Mandatory emergency management training as per practice accreditation process; high standards of work health & safety requirements for accreditation with specialised agencies.
	(1b) In-service guidance	Individualised practice guidelines regarding disaster screening, detection and management. Mandated training & education for staff on disaster preparedness and management Multidisciplinary training with various PHCPs to prepare for a cohesive disaster response.
2. Established Communication Channels	(2a) External communication with PHCPs	Emails or faxes from primary healthcare networks regarding updated regulations and guidelines during disaster response.
	(2b) In-service communication	Regular staff meetings and email correspondence to establish updated policies and guidelines, particularly during disasters and high-risk seasons. Strong in-service support network for PHCPs to contact regarding any concerns, queries and recommendations regarding disaster management.
	(2c) Communication between PHCPs and their community	Patient education on disaster prevention and management via phone calls, flyers, brochures and posters.
3. Resources for Acute Disaster Response	(3a) Material resources	Basic in-service emergency resource supply available for acute emergency response Provision of disaster preparedness and management resources from primary healthcare networks during disasters. Increased availability of in-service resources for disaster prevention, screening and management during disaster-prone seasons.
	(3b) In-service personnel	Flexible working hours to increase workforce during emergency response. Flexible surge capacity to accommodate for staff sickness or absence during disaster response. Increase surge capacity during disasters to share patient load with nearby PHCPs to meet increasing demand for primary healthcare during disaster response and recovery.
	(3c) Knowledge	Access to recommendations, policies, and guidelines from local and international disaster responses to be integrated into pre-existing contingency plans.
4. Moral Obligation	Personal accountability to seek and attend additional disaster management courses and upskilling workshops. Duty of care to maximise preparedness by attending regular disaster readiness training. Duty of care for PHCP services to maintain supply of emergency resources.	
5. Digital Technology Facilitating Business Continuity	Transition from paper to electronic data, allowing a safer, more reliable platform to access information. Automatic backup and restoration of electronic data during power outages. Back-up power supply to maintain access to computer hardware & monitor vaccine refrigerators at optimal storage temperatures.	
6. Continuity of Care	Strong patient rapport facilitating the delivery of patient and community education on disaster management (e.g., disaster prevention measures; tackling vaccine hesitancy). Community trust in PHCPs facilitating effective decision-making during disaster prevention and response (e.g., vaccinations). Strong patient rapport enabling PHCPs to build and use the local knowledge of the community to deliver psychosocial support.	

**Table 3:** Internal and external barriers to PCHPs’ involvement in disaster management.

Category	Subcategory	Theme
1. Lack of understanding & recognition of the role of PHCPs	(1a) Primary health networks	No defined duty, role or response of PHCPs in disaster management guidelines, which outline a predominantly hospital-based response. Limited involvement of PHCPs in disaster planning and preparation, leading to insufficient use of the full capacity & resources of PHCPs during disaster response.
	(1b) Community	Limited community understanding of the role of PHCPs in facilitating unneeded presentations to tertiary hospitals during disasters.
2. Lack of resources	(2a) From governments and agencies	Insufficient governmental funding for material resources for disaster response, particularly in prolonged disasters. Insufficient federal funding to ensure personal safety for PHCPs during disaster response. Limited availability of community mental health services due to limited understanding of mental health and prevention measures.
	(2b) Internal workforce	Lack of staff availability, particularly during the recovery stages of disaster. Conflicting balance between work, training and external commitments during disaster response and recovery. Resource-intensive to organise regular, hands-on in-service training sessions.
3. Lack of interest in disaster management	Lack of foreseeable benefit of disaster preparedness due to the low recurrence of disasters. Lack of general awareness of the repercussions of disasters. Unfeasibility to be maximally prepared for all types of potential disasters. Not following contingency plans from previous disasters.	
4. Lack of renumeration	Lack of financial incentives to partake in additional training and education workshops. Lack of additional incentives to increase work hours such as financial remuneration of accreditation of training.	
5. Lack of comfort and self-perceived competence	Lack of previous encounters and experience in disaster management. Limited clinical training or hands-on exposure for upskilling, and lack of recognition cause diminished confidence for PHCPs to be involved at a higher capacity in disaster management. Staff hesitation to work due to high risk to self and personal safety during disaster response.	

## Data Availability

On reasonable request, the corresponding author can provide access to the data used to support the study’s findings.

## Appendix A. Consolidated Criteria for Reporting Qualitative Research (COREQ)

**Domain 1: Research Team and Reflexivity**				
**Section**		**Item**	**Guide Questions/Description**	**Comment**
Personal characteristics	1	Interviewer/facilitator	Which author/s conducted the interview or focus group?	DL, SG, KS
	2	Credentials	What were the researcher’s credentials?	EA-MB SA-MD HM-PhD RS-MPH SG-MD KS-MD KW-MD GK-MBBS DL-DrPH
	3	Occupation	What was their occupation at the time of the study?	DL, GK: supervisors EA, RS, SG, KS: student-researchers SA, HM, KW: external validation
	4	Gender	Was the researcher male or female?	EA, SA, SG, GK, DL: male HM, RS, KS, KW: female
	5	Experience and training	What experience or training did the researcher have?	SA, SG, KS, KW, GK, DL: clinician-researchers EA, RS: health service HM: social scientist
Relationship with participants	6	Relationship established	Was a relationship established prior to study commencement?	RS, SG, KS, KW were students of DL EA is PhD candidate supervised by SA, HM and DL
	7	Participant knowledge of the interviewer	What did the participants know about the researcher?	GK was on the Royal Australian College of General Practitioners committee that developed pandemic resources, but all participants were not personally known to GK, and GK was not involved in data collection.
	8	Interviewer characteristics	What characteristics were reported about the interviewer/facilitator?	Participants were aware of the intent of this student-led project and may have investigated the reputation and trustworthiness of the supervisor DL. Participants were not remunerated for their time or involvement.
